# Adipose Tissue Dysfunction as Determinant of Obesity-Associated Metabolic Complications

**DOI:** 10.3390/ijms20092358

**Published:** 2019-05-13

**Authors:** Michele Longo, Federica Zatterale, Jamal Naderi, Luca Parrillo, Pietro Formisano, Gregory Alexander Raciti, Francesco Beguinot, Claudia Miele

**Affiliations:** 1 Department of Translational Medicine, Federico II University of Naples, 80131 Naples, Italy; mi_longo@libero.it (M.L.); federicazatterale@libero.it (F.Z.); jamal.naderi83@gmail.com (J.N.); lparrillo@alice.it (L.P.); fpietro@unina.it (P.F.); gregoryraciti@gmail.com (G.A.R.); 2 URT Genomic of Diabetes, Institute of Experimental Endocrinology and Oncology, National Research Council, 80131 Naples, Italy

**Keywords:** obesity, adipose tissue, lipotoxicity, insulin resistance, diabetes, hypertrophic obesity, inflammation, adipogenesis, ectopic lipid deposition, adipose tissue dysfunction

## Abstract

Obesity is a critical risk factor for the development of type 2 diabetes (T2D), and its prevalence is rising worldwide. White adipose tissue (WAT) has a crucial role in regulating systemic energy homeostasis. Adipose tissue expands by a combination of an increase in adipocyte size (hypertrophy) and number (hyperplasia). The recruitment and differentiation of adipose precursor cells in the subcutaneous adipose tissue (SAT), rather than merely inflating the cells, would be protective from the obesity-associated metabolic complications. In metabolically unhealthy obesity, the storage capacity of SAT, the largest WAT depot, is limited, and further caloric overload leads to the fat accumulation in ectopic tissues (e.g., liver, skeletal muscle, and heart) and in the visceral adipose depots, an event commonly defined as “lipotoxicity.” Excessive ectopic lipid accumulation leads to local inflammation and insulin resistance (IR). Indeed, overnutrition triggers uncontrolled inflammatory responses in WAT, leading to chronic low-grade inflammation, therefore fostering the progression of IR. This review summarizes the current knowledge on WAT dysfunction in obesity and its associated metabolic abnormalities, such as IR. A better understanding of the mechanisms regulating adipose tissue expansion in obesity is required for the development of future therapeutic approaches in obesity-associated metabolic complications.

## 1. Introduction

Severe obesity is associated with elevated risks of adverse health consequences. The prevalence of obesity is rising worldwide, and if the trend continues, global prevalence will reach 18% in men and 21% in women by 2025 [1]. A positive energy balance between energy intake and energy expenditure results in weight gain and obesity [2]. Many factors, including genetics, epigenetics, and lifestyle factors, have been implicated in obesity pathogenesis [2,3,4,5,6,7]. In most cases, no single factor is exclusively responsible for the development of obesity. Rather, obesity results from the interaction of these factors and these combinations can vary over time and between individuals [2,3,4]. Dietary and lifestyle interventions can be adequate to treat obesity and prevent metabolic alterations. Moderate and progressive weight loss improves metabolic function in different tissues and contributes to dose-dependent changes in the main adipose tissue biological pathways. Nevertheless, these approaches are difficult to maintain in the long term [8].

Obesity is a critical risk factor for the development of type 2 diabetes (T2D). By 2025, more than 300 million people are expected to have T2D as a complication of obesity [9]. The primary cause of T2D is obesity-driven insulin resistance (IR) in white adipose tissue (WAT), liver, and skeletal muscle, combined with impaired secretion of insulin by pancreatic β-cells to overcome this resistance [10]. Obesity-induced IR is also linked to a wide cluster of obesity-associated metabolic abnormalities, such as dyslipidemia, non-alcoholic fatty liver disease (NAFLD), hypertension [11], coronary heart disease, and stroke [12].

Insulin reduces blood glucose by inducing glucose uptake in insulin-sensitive tissues (skeletal muscle, adipose tissue, and liver) and by inhibiting glucose production in liver. IR occurs when the insulin-sensitive tissues lose insulin response. In this scenario, insulin-mediated glucose uptake is impaired in the insulin target tissues. This failure is a result of the insulin signaling pathway inhibition [13]. Nonetheless, an overall paradigm has been strengthened by many studies over several decades [14,15] in which overnutrition in predisposed individuals leads to IR in peripheral tissues. This effect increases blood glucose levels, which in turn stimulates the β-cell insulin secretion [16]. There are several hypotheses to explain the mechanisms responsible for IR in obese subjects. These mechanisms include adipose tissue dysfunction/lipotoxicity, inflammation, mitochondrial dysfunction, hyperinsulinemia, and endoplasmic reticulum (ER) stress. Although there is no theory for a unifying mechanism, most of these factors are typically and concomitantly associated with obesity. Here, we review the current knowledge of WAT dysfunction in obesity and its associated metabolic abnormalities.

WAT is a complex organ and has primary roles in energy homeostasis control. Adipocytes not only act as a reservoir for energy storage and utilization, but also sense energy demands and secrete paracrine factors to regulate other metabolic tissues. In a high energy state, for example, leptin is secreted from adipocytes to reduce food intake centrally and increase energy expenditure [17,18]. However, in obesity, WAT may become severely dysfunctional and not expand properly to store the energy excess. This induces ectopic fat deposition in other tissues that regulates glucose homeostasis, an event commonly defined as “lipotoxicity”. This mechanism leads to systemic IR and an increased risk of T2D [19,20]. Numerous deleterious effects have been associated with the unhealthy expansion of the WAT, including inflammation, fibrosis, hypoxia, altered adipokines secretion, and mitochondrial dysfunction, each of which could represent a new therapeutic target in the obesity treatment [10]. In prolonged positive energy balance conditions, adipocytes expand cell size and number to compensate the need for increased lipid storage. These cells inevitably reach a limit at which additional anabolic pressure cannot be accommodated, due to cell and tissue expansion limitations. Reaching this threshold causes stress in adipocytes and initiates an inflammatory program in response to this stress [19].

**Figure 1:**
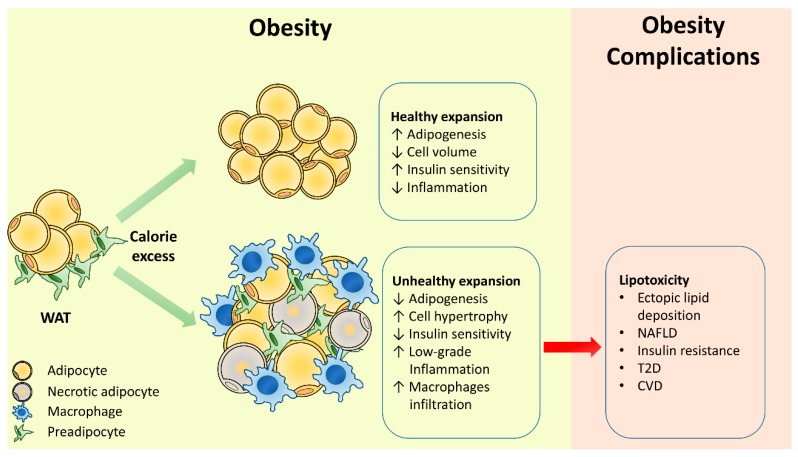
White adipose tissue expansion in obesity. White adipose tissue responds to caloric excess through a healthy or unhealthy expansion. Healthy expansion through adipocyte hyperplasia protects against the metabolic complications of obesity. Unhealthy expansion through adipocyte hypertrophy promotes the obesity-associated metabolic complications. WAT, white adipose tissue; T2D, type 2 diabetes; NAFLD, non-alcoholic fatty liver disease; CVD, cardiovascular disease.

In obesity, “healthy” WAT expansion is achieved by recruiting and differentiating adipose precursor cells rather than infiltrating fat into mature adipocytes. Alterations in the precursor cell commitment and subcutaneous adipose tissue (SAT) adipogenesis are associated with the metabolic complications of obesity. When the storage capacity of SAT, the largest adipose tissue depot, is exceeded, further caloric overload leads to the fat accumulation in ectopic tissues (liver, skeletal muscle, and heart) as well as in the visceral depots. It has been largely demonstrated that excessive lipid accumulation in ectopic tissues leads to local inflammation and IR (Figure 1). The ectopic fat accumulation in the pancreas, for example, contributes to β-cell dysfunction, and recent studies in human have proved that the bariatric surgery can improve β-cell function by decreasing pancreatic fat accumulation [21,22]. A marker of ectopic fat accumulation in human is the increased visceral/intra-abdominal fat accumulation, associated with abdominal obesity [23]. Independently of body mass index (BMI), adipose tissue dysfunction, increased visceral and ectopic fat accumulation, and inflammation may contribute to unhealthy obesity and associated IR.

Although IR has, by definition, different potential pathogenic mechanisms, we believe that, given the relevance of its association with obesity, it is likely that adipose tissue dysfunction becomes the major contributor to subsequent associated complications in a high percentage of obese patients. This review outlines the current knowledge on WAT expansion in obesity and highlights the mechanisms that make it dysfunctional and associated with metabolic alterations, including inflammation, impaired adipogenesis, and ectopic lipid deposition.

## 2. Adipose Tissue Remodeling in Obesity

The adipose tissue has a crucial role in the regulation of systemic energy homeostasis acting as a “safe” depot to store excess fat. In overnutrition, mature adipocytes accumulate more fat and undergo cellular hypertrophy [24], whereas during calorie restriction they provide nutrients to other tissues through lipolysis [25].

To review the adipose tissue remodeling in obesity and associated metabolic comorbidities, it is essential to examine how the morphology can change depending on the adipose tissue location. The adipose tissue is classified, according to the regional distribution, as SAT (located under the skin) and visceral adipose tissue (VAT; associated with internal organs), and it is diffused throughout the entire human body [26]. The sites of adipose tissue accumulation are strictly conserved across several species [26,27,28,29]. The development and formation of these two adipose tissue types are different, and even in adult life, they show different functions and structures [30,31]. Adiposity is a polygenic trait; several genes control phenotypic variability [32], and multiple pathways regulate its development [33].

Different studies report that fat distribution is strongly associated with IR, the main risk factor for T2D and cardiovascular disease (CVD) [34]. A systematic review and meta-analysis of observational studies by Zhang et al. demonstrates that the accumulation of VAT is the strongest predictor of IR [35]. Nevertheless, obesity indices (total fat mass, BMI, and waist circumference) and adipose tissue depots (intra-abdominal and total abdominal fat) are significantly correlated with IR [35]. Other human studies have also shown that the accumulation of lipids in the abdominal SAT correlates with the onset of IR and T2D. Central adiposity rather than peripheral adiposity is an important risk factor in establishing metabolic diseases [36,37].

In response to a positive energy balance, dynamic mechanisms reorganize the adipose tissue by changing the number and size of mature adipocytes. In the meantime, the precursor cells of the stromal vascular fraction begin to be recruited and committed towards the adipocyte lineage. Hypertrophic adipocytes secrete paracrine factors (hormones and cytokines), which facilitate preadipocytes recruitment and promote their differentiation into mature adipocytes [38]. These events are generally defined as “adipose tissue remodeling” [39]. In obesity, alteration in adipose tissue remodeling may induce the dysregulation of adipose tissue secreted cytokines, leading to local and systemic inflammation and impaired adipogenesis of precursor cells, as further discussed later in this review [40,41].

In addition to the regional distribution of fat, the adipocyte morphology (hypertrophy vs. hyperplasia) contributes to the obesity-associated metabolic abnormalities. In a chronic state of positive energy balance, the adipocyte size reaches a critical threshold before recruiting precursor cells to increase the adipocytes number. Spalding et al. demonstrated that the adipocyte number is tightly regulated and determined during childhood, suggesting that the increase in cell size is the main plasticity mechanism in response to an energy imbalance [42]. Adipose tissue hyperplasia is considered as a “recovery mechanism” to overnutrition [42]. The adipocytes that reach the critical cell size become lipid-overloaded and insulin-resistant, and adipose tissue hyperplasia attempts to repair metabolic alterations [43]. In vivo data confirm these observations in AdipoChaser mice, a model to track adipogenic footpath in vivo [44]. In diet-induced obesity, AdipoChaser mice already show hypertrophic VAT in four weeks, while tissue hyperplasia occurs within two months. Interestingly, SAT exhibits only hypertrophy by two months of high-fat diet and limited adipogenesis. Using stable isotope methodology to measure SAT and VAT adipogenesis, Kim et al. confirmed these observations and found a positive association between adipocyte turnover and insulin sensitivity. They identified adipocyte hypertrophy as the major mechanism of adult fat mass expansion, supporting the concept that the failure of adipose tissue plasticity results in IR and metabolic disease [45]. Similar findings have also been reported in human study [46].

The impaired adipose tissue remodeling in obesity is not a homogeneous condition, and obesity does not necessarily translate into IR and increased risk for metabolic comorbidities. Several studies have reported that a subgroup of obese individuals remains insulin-sensitive and metabolically “healthy” and exhibits normal physiology and hormonal profiles [47,48,49]. Such healthy but overweight individuals are classified as “metabolically healthy obese” [50]. They exhibit increased subcutaneous adiposity but reduced adipose inflammation and expansion of VAT. Nevertheless, longitudinal studies are providing compelling evidence that metabolically healthy obesity is likely to be a transient condition [51,52]. Furthermore, to support this concept, a part of the obesity spectrum is represented by metabolically obese normal-weight (MONW) individuals [53,54]. Thirty years ago, Ruderman et al. introduced the concept that some non-obese individuals show several risk factors (increased adipose cell size and hyperinsulinemia) for metabolic disorders [55]. Investigations revealed that these MONW individuals are characterized by increased levels of visceral adiposity, IR, and a higher susceptibility to T2D and CVD [56]. These data indicate that both regional depositions of adipose tissue (visceral and/or subcutaneous) and adipocyte morphology (cell size; hypertrophy and/or hyperplasia) contribute to an increased risk of IR [38,57]. In line with these findings, individuals with increased adipogenic capacity in SAT display a reduced adipose cell size and maintain a healthy metabolic state. In a cohort of unhealthy obese subjects, the adipocyte volume threshold predicts an increased risk for obesity-associated T2D. An increased adipocyte size is also associated with a lower improvement of IR after bariatric surgery. Moreover, lipid-overloaded hypertrophic adipocytes per se are sufficient to cause IR in adipose tissue [defects in glucose transporter type 4 (GLUT4) trafficking to the plasma membrane], independently of adipocyte inflammation [58,59]. However, other studies place inflammation at the center of the mechanisms by which hypertrophy leads to IR, as further discussed below. Increased pro-inflammatory cytokines [tumor necrosis factor alfa (TNF-α), interleukin-6 (IL-6), interleukin-8 (IL-8), and monocyte chemoattractant protein1 (MCP-1)] secretion [60] by hypertrophic adipocytes leads to serine phosphorylation of insulin receptor substrate-1 (IRS-1), therefore preventing the insulin signaling [61]. Pro-inflammatory cytokines also promote local and systemic inflammation by recruiting macrophages and T-cells [41,62]. Hypertrophy also induces local adipose tissue hypoxia [63] that activates hypoxia-inducible factor (*HIF*) 1α, increases the local inflammation and accelerates adipose tissue fibrosis [64].

Furthermore, hypertrophic adipocytes manifest significant alterations in cell metabolism. Basal lipolysis is elevated in hypertrophic adipocytes [65], increasing the leakage of free fatty acids (FFAs). Conversely, smaller insulin-sensitive adipocytes show a higher lipogenesis-to-lipolysis ratio [66]. In unhealthy obesity, fat mobilization from adipocytes is impaired, and insulin is unable to suppress lipolysis. Unesterified fatty acids and cholesterol spill over from large adipocytes into ectopic sites that are not designed primarily for lipid storage. This mechanism is a major trigger of lipotoxicity and systemic IR [20,67,68]. Several criteria have been adopted to define hypertrophic or hyperplastic WAT. In 2010, Arner et al. defined a morphology value as the difference between measured adipocyte volume and expected adipocyte volume (based on a curvilinear relationship between fat cell volume and fat mass in 764 subjects) [42,69]. A positive value indicates an adipocyte volume larger than expected and subjects were classified as hypertrophic, while a negative value indicates an adipocyte volume smaller than expected and subjects were classified as hyperplastic. In a cohort of 764 subjects, Arner et al. found that the occurrence of hyperplasia or hypertrophy is independent of sex and body weight but correlates with fasting plasma insulin levels and insulin sensitivity. The total adipocyte number and morphology are negatively related, and the number of total adipocytes is increased in hyperplasia than in hypertrophy. The total number of newly generated adipocytes each year is 70% lower in hypertrophy than in hyperplasia [69].

Other studies have concluded that adipocytes include a heterogeneous population of cells that display a bimodal distribution based on their cell size. Measurement by microscopy highlights a peak of small adipocytes of ∼25 μm in diameter and a peak of larger adipocytes of ∼50 μm in diameter [57,70]. The size of the larger fraction of the adipocytes in a bimodal distribution is positively associated with metabolic dysfunction. McLaughlin et al. showed that insulin-resistant individuals had larger adipocytes (50 μm fraction) in the abdominal SAT when compared with insulin-sensitive individuals [70]. Furthermore, an increase in the size of the larger adipocytes fraction after feeding a high-fat diet predicts deterioration of insulin-stimulated glucose uptake in insulin-sensitive obese individuals [24]. Alterations in the adipose tissue plasticity are the major trigger of the obesity-associated metabolic complications. In obesity, the inadequate fat depots response to the caloric overflow leads to systemic metabolic alterations.

## 3. Impaired Adipogenesis and Insulin Resistance in Adipose Tissue Dysfunction

The limited expandability of the SAT leads to an inappropriate adipose cell expansion with local inflammation and insulin-resistant phenotype [71]. By contrast, the expansion of adipose tissue by enhanced adipogenesis not only distributes excess fat between newly differentiated adipocytes, but also reduces the number of hypertrophic adipocytes that secrete inflammatory cytokines [72]. Promoting adipose cell recruitment in the SAT rather than merely inflating the cells would be protective from the obesity-associated metabolic complications.

**Figure 2:**
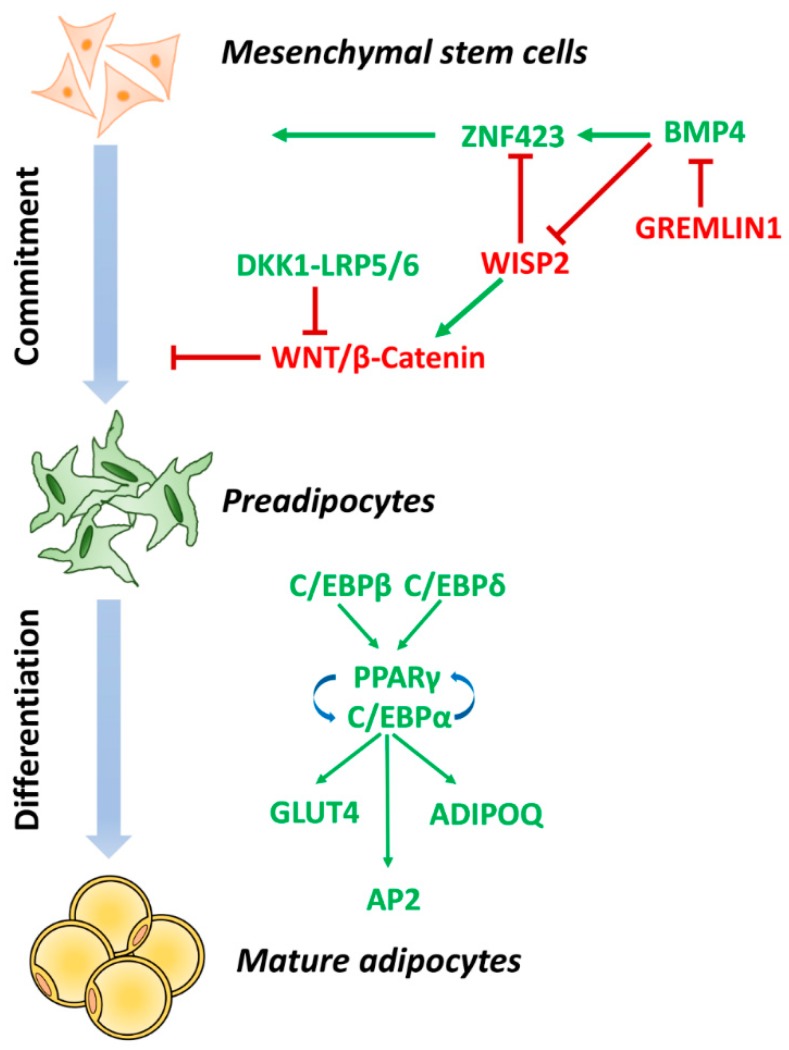
Molecular mechanisms of adipogenesis. Adipogenesis can be divided into two phases: commitment and terminal differentiation. Bone morphogenetic protein 4 (BMP4) induces mesenchymal progenitor cells to the adipocyte lineage as part of the adipogenic commitment. As a result of BMP4 signaling pathways, committed preadipocytes express the transcriptional activator zinc-finger protein 423 (*ZNF423*), a fundamental determinant of preadipocyte cell fate. The commitment phase also requires coordinated inhibition of several pathways [e.g., wingless-type mouse mammary tumor virus integration site family (WNT) and wnt1-inducible-signalling pathway protein 2 (WISP2)]. During terminal differentiation phase, committed preadipocytes arrest their growth and activate early differentiation markers, including peroxisome proliferator-activated receptor-γ (*PPARγ*) and transcription co-activators CCAAT/enhancer-binding protein α (*C/EBPα*). C/EBPα and PPARγ induce and maintain the expression of key adipogenic genes (*GLUT4*, *AP2*, and *ADIPOQ*), which are necessary for normal adipocyte function. WISP2, WNT1-inducible signaling pathway protein 2; DKK1, proadipogenic factors Dickkopf 1. LRP5/6, lipoprotein-receptor-related protein-5 or -6; GLUT4, glucose transporter type 4; AP2, adipocyte fatty acid-binding protein 2.

Pluripotent mesenchymal stem cells (MSCs) can develop into several cell types, including adipocytes, myocytes, chondrocytes, and osteocytes. These stem cells are located in the vascular stroma of adipose tissue as well as in the bone marrow [73]; indeed, bone marrow-derived cells account for approximately 10% of the SAT cell population and are therefore increased by up to 25% in obese people [74]. MSCs, when appropriately stimulated, undergo a multistep process of commitment in which the progenitor cells become restricted to the adipocyte lineage [73]. Accordingly, adipogenesis can be divided into two phases: commitment (or determination) and terminal differentiation. Determination results in the conversion of the stem cell into a preadipocyte, which cannot be distinguished morphologically from its precursor cell but has lost the potential to differentiate into other cell types. In the second phase, the terminal differentiation, the preadipocyte takes on the characteristics of the mature adipocyte that acquires the necessary machinery for lipid transport and synthesis, insulin sensitivity, and the secretion of adipocyte-specific proteins. All of these steps are controlled by a network of interacting transcription factors operating to coordinate the expression of many hundreds of proteins responsible for establishing the mature fat cell phenotype (Figure 2) [75,76].

### 3.1. Adipocyte Commitment

The wingless-type mouse mammary tumor virus integration site family (WNT) signaling pathway is a fundamental regulator of preadipocytes commitment. WNTs family ligands are the secreted glycoproteins that regulate adult tissue homeostasis and remodeling by autocrine and paracrine mechanisms [77]. WNTs exert their effects by signaling through “canonical” and “non-canonical” pathways to control cell proliferation, survival and determination. Canonical WNT pathway activation results in stabilization and translocation of the β-catenin into the nucleus. In preadipocytes, this results in a failure induction of peroxisome proliferator-activated receptor gamma (*PPARγ*), CCAAT/enhancer-binding protein alfa (*C/EBPα*) and a shift towards an osteoblastic cell lineage [72,77,78]. The WNT signaling pathway thus plays a critical role in maintaining uncommitted and undifferentiated precursor cells and its termination is a prerequisite for allowing for induction of adipogenic differentiation. Dickkopf (DKK) family proteins specifically inhibit the canonical WNT pathway by binding as an antagonist to the low-density lipoprotein-receptor-related protein-5 or -6 (LRP5/6) co-receptors. Expression of *DKK1* gene and protein is transiently induced and secreted during differentiation of human preadipocytes as an autocrine regulator. This leads to the inhibition of WNT signaling pathways and induction of preadipocytes commitment and differentiation [77]. However, there is an impaired inhibition of the canonical WNT pathway in hypertrophic obesity, partially due to a failure induction of *DKK1* gene expression in adipocyte precursor cells [79,80]. Nevertheless, the inhibition of canonical WNT signaling through DKK1 secretion is not sufficient to induce adipogenic commitment of the preadipocytes, as this requires coordinated activation and/or inhibition of several other pathways.

Several studies have identified that bone morphogenetic protein 4 (BMP4) is sufficient to drive adipocyte commitment and is required for adipogenic differentiation in vitro. BMP4 binds its receptor and signals to activate the downstream transcription factor SMAD Family Member 4 (SMAD4) [81,82]. The activated SMAD4 is then able to induce terminal differentiation in preadipocytes by stimulating transcription of *PPARγ*, the key regulator of adipogenesis. Indeed, BMP4 induces nuclear entry of the PPARγ transcriptional activator zinc-finger protein 423 (ZNF423) [83], through the dissociation of an intracellular protein complex between wnt1-inducible-signalling pathway protein 2 (WISP2) and ZNF423 which retains ZNF423 in the cytosol. BMP4 dissociates this complex, allowing nuclear entry of ZNF423, thereby activating *PPARγ* transcription and commitment of precursor cells into the adipocyte lineage [83]. Many human studies show numerous alterations in this pathway in subjects characterized by SAT hypertrophy with an inability to recruit and differentiate preadipocytes [79,82,83]. BMP4 is highly expressed and secreted by adipocytes, in particular in obese individuals [82]. This is probably a feedback signal to recruit new cells in order to prevent further pathologic expansion of adipose cells. Nevertheless, this does not work in hypertrophic obesity; indeed, secretion of BMP4 antagonists, in particular Gremlin 1 in humans, is increased in hypertrophic obesity and prevents the expected positive effect of BMP4 on adipogenesis [82]. The inability to dissociate WISP2/ZNF423 complex favors the development of hypertrophic obesity and associated metabolic consequences, including IR and T2D. In addition, Smith and colleagues [83] have identified the WISP2 protein as a novel adipokine involved in the crosstalk between WNT and BMP4 signaling pathways. WISP2 protein is highly expressed in early adipogenic precursor cells and the SAT of individuals with hypertrophic obesity. WISP2 acts both intra- and extracellularly [79,84] and has the potential to enhance the proliferation of MSCs in WAT [85]. Secreted WISP2 is an atypical WNT ligand, which activates the canonical WNT pathway through an unidentified signaling pathway, which involves LRP5/6 co-receptor. This prevents the adipogenesis process and allows the cells to proliferate and remain lineage-uncommitted [79]. Inside the cytosol, WISP2 protein forms a complex with the regulator of preadipocyte determination ZNF423, preventing its translocation into the nucleus and the ZNF423-mediated upregulation of *PPARγ* gene [83]. The zinc-finger transcription factor Zfp423 (mouse orthologue) has been identified as a fundamental determinant of preadipocyte commitment [86]. *Zfp423* ectopic expression in non-adipogenic cells is sufficient to activate *PPARγ* expression and increase the adipogenic potential of these cells, while its knockout impairs the development of white and brown adipose tissue in mice [86]. Thus, Zfp423 is crucial for the initial formation of WAT and plays an important role in maintaining the energy-storing phenotype of white mature adipocytes at a later stage [87].

We have recently demonstrated [88] that changes in DNA methylation at the *ZNF423* gene promoter are key mechanisms in the regulation of its transcription, and these epigenetic events are fundamental to enable precursor cells to differentiate into mature adipocytes. Furthermore, our results in human preadipocyte reveal that the expression of *ZNF423* negatively correlates with the cell size of human subcutaneous adipocytes. In hypertrophic obese individuals, a massive hypermethylation occurs at CpG dinucleotides within a promoter region of the human *ZNF423* gene and closely correlates with the reduced *ZNF423* expression in the adipocyte precursor cells. We have also shown that BMP4 causes demethylation of the *Zfp423* promoter, which is sufficient to commit otherwise non-adipogenic cells to the adipogenic lineage. Thus, the convergence of BMP4 signaling on Zfp423 enables its action on pre-adipocyte determination through multiple mechanisms, including epigenetic modifications at key genes and nuclear translocation of Zfp423 [88]. Hence, changes in the methylation profile at a specific regulatory region of the *ZNF423* gene account for its transcription regulation and may explain the impaired adipogenesis of the preadipocytes observed in human hypertrophic obese subjects.

### 3.2. Adipocyte Terminal Differentiation 

The molecular regulation of terminal differentiation is more extensively characterized than determination because of the use of cell lines that have a restricted potential to differentiate into other cell types such as 3T3L1 and 3T3-F442A murine cells.

Adipogenesis, and in particular terminal differentiation, includes a series of transcriptional processes involving the sequential expression of several transcriptional factors, culminating in the activation of C/EBP proteins and PPARγ, the central transcriptional regulators of adipogenesis. The first step involves the temporary induction of *CEBPβ* and *CEBPδ*, which in turn directly drives the expression of *C/EBPα* and *PPARγ* [7]. C/EBPα and PPARγ functionally synergize to activate the mature adipocyte program properly. More than 90% of *PPARγ* DNA-binding sites also bind C/EBPα. These factors cooperatively orchestrate adipocyte biology by adjacent binding sites and establish the mature adipocyte phenotype [72,89]. When activated, C/EBPα and PPARγ induce and maintain the expression of key adipogenic genes, such as *GLUT4*, adipocyte fatty acid-binding protein 2 (*AP2*), and adiponectin, which are necessary for normal adipocyte function including insulin sensitivity [89].

Thiazolidinediones (TZDs), the best-known PPARγ synthetic ligands, have been used as anti-diabetic drugs, and their beneficial effects in the treatment of IR and obesity are well demonstrated. Activating PPARγ by TZDs treatment enhances WAT expansion, alleviates peripheral lipotoxicity and reduces inflammatory cytokines secretion [38]. This activation increases the WAT’s ability to store lipids and reduces ectopic lipid accumulation in the liver and muscle, by the induction of fatty acid metabolism in patients with T2D. The metabolic effects include reduced triglyceride levels in blood, liver, and muscle, coupled with increased triglyceride content in the adipose tissue [90,91].

To further support the importance of PPARγ in controlling adipogenesis and systemic insulin sensitivity, Majithia et al. identified all possible missense PPARγ variants in the normal population that impair adipocyte differentiation and that are associated with an increased risk for the onset of T2D [92]. In addition to these, other PPARγ variants have been recently recognized. Aprile and colleagues identified a truncated isoform of PPARγ (PPARγΔ5), which lacks the entire ligand-binding domain. PPARγΔ5 is expressed in human adipose tissue and, during adipocyte differentiation, acts as a dominant-negative isoform by reducing PPARγ activity and impairing the differentiation ability of preadipocytes. Additionally, *PPARgΔ5* expression in SAT positively correlates with BMI in obese and T2D patients, possibly contributing to adipose tissue dysfunction and associated metabolic alterations [93].

These findings support the hypothesis that alterations in adipose tissue expansion in obesity, caused by impaired adipogenesis, are closely associated with IR.

## 4. Chronic Inflammation Links Obesity to Insulin Resistance

To explain the pathogenesis linking obesity with IR and diabetes, several studies support a correlative and causative association between nutrient excess and activation of the innate immune system in organs involved in energy homeostasis [94]. Adipose tissue has been historically considered only a storage organ. However, this view was revised after Spiegelman’s group revealed that adipose tissue acts as an important active endocrine organ [39,95]. Adipose tissue secretes lipids, bioactive peptides (adipokines), and other metabolites, modulating whole-body energy and glucose homeostasis [39,96,97]. White adipose depots are composed of various cell types such as endothelial cells, fibroblasts, preadipocytes, stem cells, and multiple immune cells that work together to maintain adipocytes integrity and hormonal sensitivity [98]. Inflammation occurs as a consequence of obesity and recent insight suggests that it may play a causal role in inducing IR [99]. The first mechanistic evidence of the inflammatory origin of obesity and diabetes comes from human and animal investigations conducted in the early 1990s. The WAT of obese rodents and humans was found to exhibit inflammatory changes and increased levels of the proinflammatory cytokine TNF-α able to induce IR [95]. As a general observation, insulin-resistant obese individuals exhibit a high degree of adipose tissue inflammation, whereas obese patients that remain insulin sensitive show no features of tissue inflammation [100]. A sustained weight loss has been shown to ameliorate systemic glucose homeostasis by improving inflammation and insulin action in the liver [101].

As mentioned above, adipose tissue responds dynamically to alterations in calories excess through adipocyte hypertrophy and hyperplasia [18,101]. The rapid expansion of adipose tissue in obesity could provide intrinsic signals including adipocyte death, hypoxia, and mechanical stress arising from interactions between the cells and the extracellular matrix that might trigger an inflammatory response [19]. An increase in adipocyte size is accompanied by a macrophage recruitment and an elevated rate of adipocyte death. Larger adipocytes display an altered secretion of chemoattractant and immune-related genes that may promote macrophage infiltration [102]. Macrophages are the most abundant leukocytes in the adipose tissue of mice and humans contributing to obesity-induced inflammation. During obesity, they constitute up to 40% of all adipose tissue cells [103]. An increase in macrophage numbers has been found in the WAT of obese mice and human subjects as a consequence of the rising levels of several factors (e.g., FFAs, cholesterol, and lipopolysaccharide) [103].

Adipose tissue macrophages (ATMs) are classified into two major subtypes: M1, activated macrophages with proinflammatory properties, and M2, activated macrophages associated with an anti-inflammatory profile [104]. In healthy lean animals, ATMs are dispersed throughout WAT and display an activated anti-inflammatory M2 phenotype [105,106]. Macrophages in association with T regulatory cells release a cascade of anti-inflammatory mediators contributing to maintaining insulin sensitivity in adipocytes and inhibiting the dysregulation and inflammation of the adipose tissue [107]. In obesity, hypertrophic adipocytes exhibit many peculiar features, such as some necrotic-like abnormalities [108]. It has been shown that an increase in dead adipocytes prevents adipose tissue function and induces inflammation [39].

A massive influx of monocytes was observed in the adipose tissue around necrotic adipocytes where differentiate into proinflammatory M1 macrophages, forming a “crown-like structure”. M1-polarized macrophages secrete a variety of inflammatory cytokines (e.g., interleukin 1 beta (IL-1β), MCP-1, TNF-α, and IL-6) contributing to local and systemic inflammation and IR [105,109]. These proinflammatory macrophages also release chemokines to recruit the next wave of incoming monocytes. Besides macrophages, many other immune cells (e.g., dendritic cells, mast cells, neutrophils, B cells, and T cells) reside in adipose tissue during obesity, playing a key role in the development of inflammation and IR [103,110].

Adipocyte hypertrophy also results in a deficiency of vasculature and local adipose tissue hypoxia [64,111]. Hypoxia is an important trigger for the induction of adipose tissue inflammation. Several evidences indicate that hypoxia develops as WAT expands owing to a relative reduction in perfusion of the hypertrophic adipocytes or an increase in oxygen utilization [64]. Cellular hypoxia may start inflammation by inducing the *HIF-1α* gene program [112]. Exposure of WAT to hypoxic conditions can induce upregulation of many inflammatory genes [113] whereas adipocyte-specific *HIF-1α* deletion prevents obesity-induced inflammation and IR [64]. Conversely, activation in ATMs of *HIF-2α*, another key player in the hypoxic responses, has been shown to alleviate adipose tissue inflammation and IR [114].

In addition to an altered adipokine secretion, hypertrophic adipocytes show enhanced basal lipolysis, increasing the leakage of FFAs [65,115]. Secretion of these factors triggers multiple inflammatory signaling pathways in both macrophages and adipocytes. For instance, FFAs can promote inflammation by binding to toll-like receptors 2 and 4 through the adaptor protein fetuin-A, resulting in activation of nuclear factor-kappa B (NF-κB) and c-Jun N-terminal kinase (JNK) signaling pathways [104,116]. Once activated, these pathways can increase the synthesis and secretion of many chemokines (e.g., MCP-1) in adipocytes, contributing to IR and proinflammatory macrophage infiltration. For this, JNK and NF-κB are considered crucial for inflammation-induced IR [117]. JNK stress kinase induces inhibitory (serine/threonine) phosphorylation of the IRS proteins. In detail, both JNK and NF-κB pathways can phosphorylate IRS1 on serine-307 residue [118]. Inhibitory IRS1 phosphorylation impairs insulin tyrosine-phosphorylation, reducing its interaction with phosphatidylinositol 3-kinase (PI3K) [118]. A previous study conducted in high-fat diet-fed rats have shown that JNK inhibition may attenuate IR, improve insulin sensitivity, increase insulin-stimulated IRS1 tyrosine phosphorylation, and decrease IRS1 serine phosphorylation [119].

As mentioned above, NF-κB is a signaling pathway implicated in inflammation-induced IR. In physiological conditions, NF-κB proteins are sequestered in the cytoplasm by a family of inhibitors called inhibitors of κB (IκBs) [120]. Activation of IKK kinase complex induces proteasomal degradation of IκBα, leading to the NF-κB nuclear translocation. This results in the enhanced expression of several NF-κB target genes with potential involvement in the pathogenesis of IR [e.g., *IL-6*, *TNF-α*, interferon gamma (*IFN-γ*), transforming growth factor beta (*TGFβ*), *MCP-1*, and receptor for advanced glycosylation end product (*RAGE*)] [117,121]. Accordingly, NF-κB-inhibiting treatments improve IR, suggesting a critical role for the NF-κB pathway in inflammation-induced IR [122,123]. Both the JNK and NF-κB pathways are also induced following ER stress and activation of the unfolded protein response [124]. In obesity, ER stress signals and unfolded protein response are widely activated. ER stress pharmacological inhibition in different tissues (e.g., liver, adipose, and brain) can reverse metabolic dysfunction [125,126].

Besides affecting insulin action, chronic low-grade inflammation alters preadipocytes differentiation into mature adipocytes. An in vitro study has shown that the exposure of pre-adipocytes to pro-inflammatory cytokines compromises adipocyte differentiation [127]. The mechanism of TGFβ action has been extensively investigated; its secretion inhibits adipogenesis by blocking the PPARγ-CEBPα transcriptional network [128]. Following an altered adipose tissue expansion in the obese, hypertrophic adipocytes release large amounts of TGFβ, further exacerbating the impaired adipogenesis [128]. However, recent insights highlight the concept that proper adipose tissue remodeling requires activation of an acute and transient inflammatory response [72].

In conclusion, chronic low-grade inflammation leads to adipose tissue dysfunction, impairing adipogenesis and insulin sensitivity. Inflammation is a finely regulated mechanism, and defects in its balance cause adipose tissue dysfunction.

## 5. Ectopic Fat Accumulation and Insulin Resistance

Ectopic fat deposition is defined as the accumulation of triglycerides in tissues not associated with adipose tissue storage, containing only small amounts of fat [129]. These alterations have been associated with adverse effects on local and systemic insulin sensitivity [24]. According to “adipose tissue expandability and spillover hypotheses”, excess fat is stored in SAT as triglycerides, but once their storage capacity is exceeded, the excess of circulating lipids will be deposited in non-adipose organs (liver, skeletal muscle, heart, and pancreas) [130]. Limited fat storage capacity is characterized by adipocyte hypertrophy, hypoxia, and a pro-inflammatory adipose tissue phenotype that can cause local and systemic IR [131,132].

Multiple genetic, environmental and behavioral factors contribute to subcutaneous versus ectopic fat deposition [133]. Recent findings indicate that dietary fat composition affects ectopic lipid accumulation and therefore IR [134,135]. Findings from previous studies provide compelling evidence that macronutrient composition plays a role in ectopic fat deposition in liver. Indeed, fatty acid and carbohydrate composition affect the fat accumulation in the liver in isocaloric diet studies [136,137].

SAT angiogenic capacity is another factor contributing to ectopic fat deposition. Impaired angiogenesis of the adipose tissue could potentially limit adipogenesis and thus contribute to metabolic dysfunction by promoting ectopic lipid accumulation. Additionally, human SAT has a considerable capillary density and angiogenic growth capacity, but this ability has been reduced by morbid obesity and adversely correlated with insulin sensitivity [138].

Ectopic fat depots can be classified according to their local and systemic potential implications. We can speculate that there are two major subtypes of ectopic fat depots, locally acting fat depots such as pericardial, perivascular, and epicardial fat, and systematically acting fat depots consisting of intrahepatic and intramuscular fat [139].

### 5.1. Liver

Liver plays a key role in maintaining hepatic fat homeostasis and energy balance through multiple metabolic pathways (e.g., de novo lipogenesis, fatty acid uptake, fatty acid oxidation, and triacylglycerol export). An imbalance between these processes could result in abnormal hepatic lipid accumulation [140,141], commonly referred to as NAFLD. NAFLD is the most frequent chronic liver disorder in the general population [142]. In people with reduced or dysfunctional SAT associated with obesity, the liver is particularly susceptible to ectopic lipid accumulation [143]. In obesity, adipose tissue is highly lipolytic and, according to the “portal hypothesis”, the liver would be directly exposed to increased levels of FFAs and inflammatory factors released from fat into the portal circulation [144].

Lipids accumulate as lipid droplets in the cytoplasm, but glycerols themselves do not damage the cells, but rather the imbalance between the above-described metabolic pathways that leads to intermediate toxic lipid synthesis (e.g., diacylglycerol and ceramides) [84,145]. Convergent evidence suggests a role in lipid-induced hepatic IR for these intermediate lipid products [146,147]. Indeed, acute ceramide depletion in adult mouse hepatocytes or adipocytes prevents and reverses hepatic lipid accumulation as well as improving systemic glucose tolerance and insulin sensitivity in diet-induced obesity mice [148]. First, both lipids have been associated with skeletal muscle IR and then assumed to mediate liver IR. However, the mechanisms proposed for hepatic IR induced by diacylglycerols and ceramides are slightly different from those identified in the skeletal muscle [148,149]. In liver, hepatic accumulation of diacylglycerides has been associated with impaired hepatic insulin signaling and IR via the induction of protein kinase Cε (PKCε), leading to a reduced insulin-stimulated phosphorylation of IRS2 and AKT serine/threonine kinase 2 (Akt2) and the ability to activate glycogen synthesis [150]. Chronic low-grade inflammation also promotes toxic intermediates accumulation in liver by increasing fatty acid uptake and triglyceride synthesis and reducing fatty acid oxidation. The anti-inflammatory therapy may improve this adverse effect [151].

A study conducted in mice overexpressing acyl-CoA diacylglycerol acyltransferase 2 (*DGAT2*) in the liver, an integral membrane protein essential for triglyceride biosynthesis, can shed light on the mechanisms [152]. Findings showed that these mice are characterized by hepatic IR associated with an increased hepatic cytosolic diacylglycerols accumulation leading to the activation of PKCε, which results in reduced IRS2 tyrosine phosphorylation and in the inability of insulin to activate hepatic glycogen synthesis and suppress hepatic glucose production [153]. These mice also exhibit a reduction in the pAkt/Akt insulin-stimulated ratio, a clear evidence of IR. In addition, they showed a slight increase in hepatic ceramide content, which may also have contributed to the hepatic IR observed in this mouse model [153]. This study reassessed the role of hepatic diacylglycerols and other lipid intermediates in causing hepatic IR in this mouse model. However, these data reflect the results found by a previous study where rats (a murine model of NAFLD), treated with DGAT2 antisense oligonucleotide, show improved hepatic insulin sensitivity which could be attributed to a reduction of hepatic diacylglycerols, triglyceride content and PKCε activation [154]. Together, these findings showed that an increase in hepatic diacylglycerol content induces PKCε activation and is responsible for the progression of hepatic IR.

### 5.2. Skeletal Muscle

Skeletal muscle, as a metabolic organ, is one of the main tissues responsible for whole-body glucose homeostasis and lipid utilization. Diacylglycerols and ceramides can also activate PKCε in the skeletal muscle in lipid over-supply conditions. Beside the liver, PKCε phosphorylates IRS1 on serine residues impairing activation of PI3K and insulin signaling in skeletal muscle [155,156]. These two lipid intermediates are directly linked to impaired insulin signaling [157].

Deletion of genes encoding for lipoprotein lipase, fatty acid transporters and DGAT1 proteins reduces skeletal muscle lipid accumulation and suppresses the above-mentioned side effects [154,158].

Increased skeletal muscle lipid content has long been considered important to induce whole-body IR in human obesity. However, insulin-sensitive and endurance-trained athletes have also increased lipid content in the skeletal muscle, coexisting with an increased oxidative capacity and lipid metabolism [159,160]. In contrast with physically inactive subjects, where lipid supply usually exceeds oxidative capacity, physically active individuals are characterized by an enhanced lipid turnover and this affects critical parameters such as the levels of specific lipid species and their cellular location [161]. Multiple evidence, therefore, suggests that it is not the total amount of intramuscular lipids per se that induces detrimental effects on the insulin sensitivity, but rather the accumulation and location of lipid intermediates [155].

### 5.3. Heart

Ectopic lipid deposition in the heart results in a form of “cardiac lipotoxicity” characterized by cardiac IR, apoptosis of the cardiac myocytes, and contractile dysfunction [162,163]. One of the earliest effects of obesity is the increased circulation of FFAs and triacylglycerols resulting in the increased fatty acid delivery to the heart [164]. The excess of fatty acids absorbed by the myocardium (referring to cardiomyocyte lipids droplets) is primarily used for energy metabolism in the mitochondria or stored as triacylglycerols [165]. However, if the mitochondrial fatty acid β-oxidation cannot match the excess fatty acid delivery due to obesity, a number of different lipid intermediates begin to accumulate, including diacylglycerols and ceramides [166]. Diacylglycerols are potent lipid second messengers that can activate several isoforms of PKC which have been implicated in the development of myocardial disease including cardiac hypertrophy and diabetic cardiomyopathy [167], whereas ceramides function as key components of lipotoxic signaling pathways linking lipid-induced inflammation and inhibition of insulin signaling [168].

The excess of fat can also be accommodated in cardiac adipose tissue. Cardiac fat is classified as epicardial adipose tissue (EAT, on the myocardium surrounding the coronary arteries), pericardial (between the visceral and parietal pericardia), and perivascular adipose tissue (PVAT, surrounding blood vessels) [169,170]. PVAT has functional relevance and implications in CVD [171]. Among its functions, PVAT influences vascular homeostasis, and in particular the contractile response. In healthy individuals, PVAT releases different vasoactive mediators able to balance the vascular function [172]. In obesity, dysfunctional PVAT leads to increased release of vasoconstrictor and pro-inflammatory molecules with subsequent changes in vascular homeostasis [173]. EAT accumulation is also crucial for the development of obesity-related CVD [174]. EAT produces a wide range of bioactive molecules in metabolic disease states. Inflammatory cytokines and reactive oxidative species, released by EAT, play a critical role in the pathogenesis of coronary artery disease and cardiac arrhythmias by developing a local proatherogenic environment [175,176]. Dietary interventions and pharmacological treatment (statin therapy) prevent EAT accumulation and promote beneficial effects on cardiac health [171,177].

## 6. Concluding Remarks

For a long time, the role of adipose tissue has been underestimated, and it has been considered a merely storage organ. The obesity pandemic has put a spotlight on adipocyte function, and we now recognize it as an endocrine organ essential in regulating systemic energy homeostasis. Obesity and the associated metabolic diseases are rapidly increasing and, in our opinion, the dysfunction of adipose tissue is the central mechanism for the development of these complications. A deep understanding of the molecular mechanisms responsible for adipose tissue dysfunction is needed. Impaired adipose tissue plasticity also synergizes with age-related metabolic defects to exacerbate metabolic disorders. Understanding the molecular alterations that regulate defective adipose tissue plasticity may identify therapeutic targets to enhance the expandability and function of adipose tissue. Lifestyle interventions as exercise and diet are effective in promoting a healthy adipose tissue expansion, although these approaches are difficult to maintain in the long term. Recently, adipogenesis has emerged as a possible therapeutic target to enhance adipose tissue health. Increasing adipogenesis during weight gain can counteract the negative metabolic consequences of obesity. However, a remaining issue is to address these mechanisms in human.

In the era of personalized and precision medicine, increasing our knowledge of adipose tissue biology might enable us to overcome the limitations of the traditional anthropometric indices of obesity. Obesity-related metabolic complications do not correlate with BMI, and additional clinical parameters are necessary for risk evaluation. There is the need to move closer to an individualized understanding of adipose tissue health and its contribution on regulating systemic energy homeostasis.

## Abbreviations

MDPI	Multidisciplinary Digital Publishing Institute
T2D	type 2 diabetes
WAT	white adipose tissue
SAT	subcutaneous adipose tissue
NAFLD	non-alcoholic fatty liver disease
BMI	body mass index
VAT	visceral adipose tissue
CVD	cardiovascular disease
MONW	metabolically obese normal-weight
GLUT4	glucose transporter type 4
TNF-α	tumor necrosis factor-α
IL-6	interleukin-6
IL-8	interleukin-8
MCP-1	monocyte chemoattractant protein 1
IRS	insulin receptor substrate
HIF	hypoxia-inducible factor
FFA	free fatty acid
MSC	pluripotent mesenchymal stem cell
WNT	wingless-type mouse mammary tumor virus integration site family
PPARγ	peroxisome proliferator-activated receptor-γ
C/EBP-α	CCAAT/enhancer-binding protein α
LRP5/6	lipoprotein-receptor-related protein-5 or -6
DKK 1	proadipogenic factors Dickkopf 1
BMP4	bone morphogenetic protein 4
WISP2	WNT1-inducible signaling pathway protein 2
TZD	thiazolidinediones
ATM	adipose tissue macrophage
NF-κB	nuclear factor-kappa B
JNK	c-Jun N-terminal kinase
PI3K	phosphatidylinositol 3-kinase
IκB	inhibitor of κB
ER	endoplasmic reticulum
PKCε	protein kinase Cε
DGAT2	diacylglycerol acyl transferase 2
EAT	epicardial adipose tissue
PVAT	perivascular adipose tissue
